# Editorial: New Insights Into Adult Neurogenesis and Neurodegeneration: Challenges for Brain Repair

**DOI:** 10.3389/fnins.2022.868876

**Published:** 2022-03-22

**Authors:** Jose A. Morales-García, Naoko Kaneko, Vicente Herranz-Pérez

**Affiliations:** ^1^Department of Cell Biology, School of Medicine, Complutense University, Madrid, Spain; ^2^Networking Research Center on Neurodegenerative Disease (CIBERNED), “Carlos III” Institute for Health, Madrid, Spain; ^3^Cellular Neurobiology Laboratory, Neurobiology Department, UCS-UCM, “Ramón y Cajal” Universitary Hospital, IRYCIS, Madrid, Spain; ^4^Nagoya City University, Nagoya, Japan; ^5^Department of Cell Biology, Functional Biology and Physical Anthropology, University of Valencia, Valencia, Spain

**Keywords:** neurogenesis, subventricular zone (SVZ), subgranular zone (SGZ), hippocampus, neural stem and progenitor cell, neurodegenerative diseases

The formation of new neurons in the brain is probably one of the most controversial topics in the scientific community since in the 1960's Joseph Altman described for the first time that proliferating cells give rise to new neurons in the adult brain of rats and other mammals. This Research Topic includes 1 brief research report, 3 mini review, 4 review and 9 original research papers gathering different contributions highlighting new developments in the field of neurogenesis.

The existence of neurogenesis in the adult has traditionally been restricted to two specific regions of the brain: the hippocampus and the subventricular zone. More recently, however, new neurons have also been found in other brain regions of adult mammals. A review by Jurkowski et al. summarizes evidence in support of the classic and novel neurogenic zones in mammals and discusses the functional significance of these new neurons. Authors also discuss the potential clinical applications of promoting neurogenesis outside of the classical neurogenic niches.

Regarding adult hippocampal neurogenesis, a fundamental problem that remains unclear is to what extent it actually occurs in humans. The answer is reviewed by Seki based on studies on adult neurogenesis performed in rodents and humans. The author also discusses the studies in non-human primates, paying attention to how data of rodent and non-human primate adult hippocampal neurogenesis should be applied for understanding this process in humans.

In contrast to mammals, the adult zebrafish brain shows neurogenic activity in a myriad of niches present in almost all brain subdivisions. In fact, the regeneration process in zebrafish after brain damage is completely different from that observed in mammals. A better understanding of that regenerative mechanisms could help to develop new therapies to combat the debilitating consequences of brain injury, stroke, and neurodegeneration. Diotel et al. review this topic to compare the properties of neural progenitors and the signaling pathways, which control adult neurogenesis and regeneration in the zebrafish and mammalian telencephalon.

The neurogenic process implies proliferation of neural stem cells and migration of precursors from the birthplace to their final site of integration. In this review, Bressan and Saghatelyan focus on the intrinsic mechanisms that regulate long-distance neuroblast migration in the adult brain and on how these pathways may be modulated to control the recruitment of neuroblasts to damaged/diseased brain areas. In this regard, intermediate progenitors, which can replenish neurons in the adult brain, have been recently identified. However, the generation of intermediate progenitors of GABAergic inhibitory neurons (IPGNs) has not been studied in detail. This original research article by Esumi et al. characterizes the spatiotemporal distribution of IPGNs in the mouse cerebral cortex. As a final part of the neurogenic process after migration, newborn neurons incorporate into existing neuronal networks throughout the lifespan, which bestows a unique form of cellular plasticity to the memory system. In this mini review article, Vergara and Sakaguchi propose several mechanisms by which hippocampal adult-born neurons could mediate memory consolidation, particularly during REM sleep, placing special emphasis on the functional correlation between new neuron activity and oscillatory dynamics in dentate gyrus and CA3 circuits.

Many factors are involved in the signaling pathways that regulate neurogenesis. This research work by Ikegaya et al. investigates the possible role of the protein netrin-5 in the stimulation and organization of the neurogenic niche in experimental animals. Similarly, estrogen and estrogen-like molecules are involved in regulating the balance between proliferation and differentiation of neural stem/progenitor cells (NSPCs), thus influencing neurogenic processes. Bustamante-Barrientos et al. provide a comprehensive literature review on the current knowledge of estrogen and estrogen-like molecules and their impact on cell survival and neurodegeneration, as well as their role in NSPCs proliferation/differentiation balance and neurogenesis.

In addition to factors, cells of the nervous system also regulate neurogenic processes. For instance, microglial cells regulate neuronal development in specialized microenvironments of the adult brain. Since recent evidence suggests that in adulthood microglia secretes factors which modulate adult hippocampal neurogenesis, Chintamen et al. discuss how interactions between immune cells and developing neurons may be leveraged for pharmacological intervention and as a means to preserve adult neurogenesis.

From a therapeutic point of view, the stimulation of adult neurogenic niches may represent a new strategy for the treatment of neurodegenerative diseases. In this respect, Longoni et al. propose in their review article that modulation of epigenetics, mitochondrial function, and neurogenesis might provide new hope for patients suffering for amyotrophic lateral sclerosis.

Animal models are so important in the study of the role of neurogenesis in neurodegenerative disorders. Clark et al. describe their results in an original research article, showing the functional importance of traumatic-brain-injuries-induced neurogenesis in assessing the potential of replacing and/or repairing hippocampal neurons in the brain after traumatic injury. In this regard, Hampton et al., using a transgenic mouse, examine the role of the molecular chaperone HspB5 in an experimental model of human tauopathy. The aim of this study was to determine whether HspB5 could present a neuroprotective effect, associated with protective responses by both microglia and astrocytes. To deepen on neurodegenerative diseases and considering recent data collected in mammalian models of multiple sclerosis, Alzheimer's disease, stroke and spinal cord injury, Vancamp et al. discuss in a review article whether thyroid hormone could have beneficial effects in various pathological contexts acting on neural stem cells.

A decline in the neurogenic process is also associated with aging. This review by Rojas-Vázquez et al. focuses on the interactions between vascular senescence, circulating pro-senescence factors and the decrease in NSC potential during aging. Understanding the mechanisms of NSC dynamics in the aging brain could lead to new therapeutic approaches, potentially including senolysis, to target age-dependent brain decline. In a similar way, Cochard et al. investigate the relationship between age-related changes in NSPC output and activity of signaling pathways downstream of the epidermal growth factor receptor, a major regulator of NSPC activity.

Adult neurogenesis is not only affected by aging and neurodegenerative diseases. Hippocampal neurodegeneration is also a consequence of excessive alcohol consumption in alcohol use disorders, being females more susceptible to alcohol-induced brain damage. In this study, Nawarawong et al. investigated the effects of alcohol dependence on NPCs in the dentate gyrus of the hippocampus and its impact on cell cycle activation during recovery in abstinence.

Finally, Yu et al. in their mini review discuss several potential drug targets that focus on the neurovascular unit informing about novel vascular-targeted therapies for neurodegenerative disorders like Alzheimer, Parkinson and amyotrophic lateral sclerosis.

Hopefully, the reader will find in this Research Topic a useful reference for the latest state-of-the-art evidence about adult neurogenesis and its implications in neurodegenerative disorders.

We thank all the authors for presenting a critical view about how our basic knowledge on neurogenesis might be used to prevent neurodegenerative disorders in the future.

## Author Contributions

All authors listed have made a substantial, direct, and intellectual contribution to the work and approved it for publication.

## Funding

This work was funded by Spanish Ministry of Science, Innovation and Universities (PCI2018-093062).

## Conflict of Interest

The authors declare that the research was conducted in the absence of any commercial or financial relationships that could be construed as a potential conflict of interest.

## Publisher's Note

All claims expressed in this article are solely those of the authors and do not necessarily represent those of their affiliated organizations, or those of the publisher, the editors and the reviewers. Any product that may be evaluated in this article, or claim that may be made by its manufacturer, is not guaranteed or endorsed by the publisher.

